# Naringenin suppresses Toll‐like receptor 2‐mediated inflammatory responses through inhibition of receptor clustering on lipid rafts

**DOI:** 10.1002/fsn3.2063

**Published:** 2020-12-16

**Authors:** Hideo Kataoka, Ayumi Saeki, Akira Hasebe, Ken‐ichiro Shibata, Takeshi Into

**Affiliations:** ^1^ Division of Oral Infections and Health Sciences Department of Oral Microbiology Asahi University School of Dentistry Mizuho Japan; ^2^ Department of Oral Molecular Microbiology Faculty of Dental Medicine and Graduate School of Dental Medicine Hokkaido University Sapporo Japan

**Keywords:** inflammation, lipid rafts, naringenin, TLR2

## Abstract

Toll‐like receptors (TLRs) are important innate immune receptors that sometimes cause excessive inflammatory responses and a perpetuated inflammatory loop that can be involved in inflammatory and autoimmune diseases. TLR2 recognizes bacterial lipoproteins in association with TLR1 or TLR6, and triggers inflammatory responses through activation of the transcription factor NF‐κB. Naringenin, a type of citrus flavonoid, has been shown to possess anti‐inflammatory properties, but its detailed action against TLR2 remains to be fully elucidated. The present study was designed to determine whether naringenin affects the inflammatory responses triggered by TLR2. Naringenin inhibited proinflammatory cytokine production and attenuated NF‐κB activation in cells stimulated with a synthetic triacylated‐type lipopeptide known as a TLR2/TLR1 ligand, as well as a synthetic diacylated‐type lipopeptide known as a TLR2/TLR6 ligand. Moreover, a similar inhibitory effect was observed in cells stimulated with a crude lipophilic fraction extracted from *Staphylococcus aureus* cell walls and in cells stimulated with *S. aureus* cells. Furthermore, we showed that such an effect is caused by inhibition of TLR2 clustering in lipid rafts on the cell membrane. These results suggest that naringenin suppresses the inflammatory responses induced by TLR2 signal transduction. Our findings indicate a novel anti‐inflammatory property of naringenin, mediated through the regulation of cell surface TLR2 functioning.

## Graphical abstract

Naringenin inhibits Toll‐like receptor 2 signaling on lipid rafts.

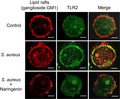

## 1 INTRODUCTION

In the innate immune system, Toll‐like receptors (TLRs) serve as the major pattern recognition receptors (PRRs) by binding with a variety of microbial components termed pathogen‐associated molecular patterns (PAMPs) (Takeda et al., 2003). During microbial infections, two cell surface TLRs, TLR2 and TLR4, are known to serve as the initial host defense mechanisms, and play a central role in initiating inflammatory responses through activation of transcription factor NF‐κB‐dependent signaling pathways (Akira & Takeda, 2004). TLR2 functions as a heterodimer, with either TLR1 or TLR6, to recognize the N‐terminal portion of bacterial lipoproteins (Ozinsky et al., 2000). The TLR2/TLR1 heterodimer recognizes common bacterial triacylated‐type lipoproteins (Takeuchi et al., 2002), whereas the TLR2/TLR6 heterodimer recognizes mycoplasmal diacylated‐type lipoproteins (Takeuchi et al., 2001). TLR4 functions with the accessory molecule MD‐2 and recognizes lipopolysaccharide (LPS), a well‐known cell wall component of gram‐negative bacteria, also called endotoxin (Shimazu et al., 1999). Along with their beneficial roles in host defense through recognition of PAMPs, these receptors also recognize endogenous ligands, termed danger‐associated molecular patterns (DAMPs). TLR2 and TLR4 have a wide range of putative endogenous ligands, including heat shock proteins, digested products of extracellular matrix, heparan sulfate, hyaluronic acid, and HMGB1 protein (van Zoelen et al., 2009). Many studies have suggested that their recognition of DAMPs causes excessive inflammatory responses and a perpetuated inflammatory loop, leading to their implication in a range of inflammatory and autoimmune diseases (Fischer & Ehlers, 2008). Therefore, effective therapeutic targeting of TLR2 and TLR4 can interrupt the inflammatory loop and ameliorate inflammatory diseases.

Botanical compounds, especially those derived from fruits, vegetables, and herbs, are known to have a wide range of beneficial effects on human health including antimicrobial, anti‐inflammatory, antioxidant, and anticancer effects (Santangelo et al., 2007). Naringenin (4′,5,7‐trihydroxyflavanone) is a flavorless and colorless flavanone, a type of citrus flavonoid, which is abundantly found in grapefruit, and is also detectable in a variety of fruits and herbs (Felgines et al., 2000). Naringenin is a potential therapeutic agent for Alzheimer's disease and has been shown to improve memory and reduce pathological accumulation of amyloid and tau proteins in mouse models (Ghofrani et al., 2015). Additionally, naringenin also demonstrates potential antimicrobial activities, and the effects of naringenin on *Staphylococcus aureus*, *Escherichia coli*, *Helicobacter pylori*, *Porphyromonas gingivalis*, and yeasts such as *Candida albicans* have been reported (Uzel et al., 2005). Furthermore, although several previous reports have shown evidence of the potential anti‐inflammatory activity of naringenin (Kawaguchi et al., 2004), it has been regarded as a subject of controversy (Gutiérrez‐Venegas et al., 2006) (Olszanecki et al., 2002). Meanwhile, recent reports have clearly demonstrated that naringenin has a potent ability to suppress the proinflammatory effects of LPS (Zaragozá et al., 2020). Interestingly, naringenin can suppress LPS‐activated TLR4‐mediated signaling, by which it ameliorates experimental colitis and endotoxemia in mouse models (Liu et al., 2016). However, it is still unclear how naringenin affects TLR2‐mediated inflammatory responses.

In this study, we examined whether naringenin exerts an anti‐inflammatory effect on TLR2‐mediated responses. We found that proinflammatory cytokine production and NF‐κB activation by TLR2/TLR1‐ and TLR2/TLR6‐stimulating ligands can be efficiently suppressed by naringenin. The results of this study indicate a novel anti‐inflammatory mechanism elicited by naringenin and suggest that it may serve as a new therapeutic agent against TLR2‐associated inflammatory diseases.

## 2 MATERIALS AND METHODS

### 2.1 Reagents and antibodies

Naringenin was obtained from Sigma‐Aldrich (St. Louis, MO, USA) and dissolved in ethanol at a final concentration of 25 mg/ml. Phorbol 12‐myristate 13‐acetate (PMA; Sigma‐Aldrich) was dissolved in dimethyl sulfoxide at a final concentration of 20 mM. A synthetic triacylated lipopeptide, Pam_3_CSK_4_ (*N*‐Palmitoyl‐*S*‐[2,3‐bis(palmitoyloxy)‐(2RS)‐propyl]‐Cys‐Ser‐Lys‐Lys‐Lys‐Lys), designed based on the N‐terminal part of the *E. coli*‐derived lipoprotein (Aliprantis et al., 1999), was purchased from Invivogen. A synthetic diacylated lipopeptide, termed FSL‐1 ((S,R)‐(2,3‐bispalmitoyloxypropyl)‐Cys‐Gly‐Asp‐Pro‐Lys‐His‐Pro‐Lys‐Ser‐Phe), designed based on the N‐terminal part of the 44‐kDa mycoplasmal lipoprotein Lp‐44 (Shibata et al., 2000) was also from Invivogen. Mouse monoclonal antibody (Ab) against human TLR2 (TL2.1) and mouse IgG2a isotype control Ab were purchased from eBioscience.

### 2.2 Cell culture

A human acute monocytic leukemia cell line, THP‐1 (RCB1189), was obtained from the Cell Engineering Division of RIKEN BioResource Center (Tsukuba, Japan). THP‐1 cells were maintained in RPMI‐1640 medium (Sigma‐Aldrich) supplemented with 10% heat‐inactivated fetal bovine serum. Human embryonic kidney (HEK) 293 cells were obtained from the American Type Culture Collection (CRL‐1573) and maintained in high glucose DMEM (Sigma‐Aldrich) supplemented with 10% heat‐inactivated fetal bovine serum. Both cells were grown at 37°C in a humidified atmosphere with 5% CO_2_.

### 2.3 Bacterial culture and extraction of a crude lipophilic fraction

*Staphylococcus aureus* ATCC 6,738 was cultivated on mannitol salt agar plates (Eiken Kagaku, Tokyo, Japan) for 3 days and then grown overnight in BBL^TM^ brain heart infusion broth (Becton Dickinson). Bacterial cells in the logarithmic growth phase were harvested by centrifugation, washed with phosphate‐buffered saline (PBS), and resuspended in PBS at an optical density (at 600 nm) of 0.5, which corresponds to approximately 4.0 × 10^7^ colony forming units (CFU)/ml.

*Staphylococcus aureus* cells were suspended in 10 mM Tris‐HCl buffer (pH 7.4) containing 150 mM sodium chloride, followed by destruction of the cell wall using sonication. The suspension was mixed with 1/10 volume of a 20% aqueous Triton X‐114 (TX; Sigma‐Aldrich) working stock solution to extract a TX‐soluble crude lipophilic fraction that mainly includes cell wall lipoproteins, as described previously (Hashimoto et al., 2006). The mixture was rotated at 4°C for 2 hr, followed by removal of cell debris using centrifugation. The supernatant was incubated at 37°C and centrifuged to separate the upper aqueous phase. An excess volume of methanol was added to the lower lipophilic phase to precipitate the lipophilic fraction at −80ºC overnight. Subsequently, the supernatant was discarded by centrifugation at 15,000 × *g* for 30 min at 4°C. The precipitated lipophilic fraction was suspended in PBS (hereafter referred to as Sa‐TX). The protein concentration of Sa‐TX was measured using a BCA assay kit (Bio‐Rad Laboratories). To investigate whether Sa‐TX contains cell‐stimulatory lipoproteins, Sa‐TX was treated with 98,100 units/ml of lipoprotein lipase (Sigma‐Aldrich) at 37°C for 6 hr.

### 2.4 Luciferase reporter gene assay

The expression vectors encoding human TLR1, TLR2, and TLR6 (pEF6‐TLR1, pEF6‐TLR2, and pEF6‐TLR6, respectively) have been described previously (Into et al., 2007; Kataoka et al., 2014). HEK293 cells were plated at a density of 1.0 × 10^5^ cells/well in 24‐well plates 1 day before transfection. The cells were transiently transfected using Metafectene**^®^** Transfection Reagent (Biontex Laboratories) with 30 ng/well of NF‐κB reporter plasmid (pNF‐κB‐Luc, Stratagene) and 3.5 ng/well of a construct directing the expression of Renilla luciferase under the control of a constitutively active thymidine kinase promoter (pRL‐TK, Promega), 150 ng/well of pEF6‐TLR2 together with 150 ng/well of pEF6‐TLR1 or pEF6‐TLR6. After a 24‐hr incubation, various concentrations of naringenin (0, 10, 20, 40 μg/ml) were added to the transfected cells together with Pam_3_CSK_4_ (10 μg/ml), FSL‐1 (10 nM), Sa‐TX (10 µg/ml), or *S. aureus* cells (4.0 × 10^7^ CFU/ml), and then incubated at 37°C in 5% CO_2_ for 6 hr. Subsequently, the cells were harvested and luciferase activity was measured using the Dual‐Luciferase**^®^** Reporter Assay System (Promega), according to the manufacturer's instructions. The relative NF‐κB activity was calculated by normalizing the NF‐κB reporter luciferase activity to the Renilla luciferase activity (as internal controls).

### 2.5 Enzyme‐linked immunosorbent assay

THP‐1 cells were plated on 24‐well plates at a density of 4.0 × 10^6^ cells/well. Subsequently, various concentrations of naringenin (0, 10, 20, or 40 μg/ml) were added to the cells together with a stimulatory compound, Pam_3_CSK_4_ (10 μg/ml), FSL‐1 (10 nM), Sa‐TX (10 µg/ml), or *S. aureus* cells (4.0 × 10^7^ CFU/ml), and incubated for 6 hr. After stimulation, the culture supernatants were collected and the amounts of interleukin (IL)‐8 and tumor necrosis factor (TNF)‐α were determined using enzyme‐linked immunosorbent assay kits for human IL‐8 and TNF‐α (PeproTech, Rocky Hill, NJ, USA), according to the manufacturer's instructions.

### 2.6 Confocal laser‐scanning microscopy

THP‐1 cells were prepared on sterilized coverslips placed in the wells of 6‐well plates at a density of 5.0 × 10^5^ cells/well and incubated in the presence of 20 nM PMA for 24 hr for differentiation into macrophage‐like adherent cells. These cells were washed three times with serum‐free RPMI‐1640, followed by treatment with *S. aureus* cells suspended in PBS (2.0 × 10^6^ CFU/ml) or PBS (as control), together with naringenin solution (40 μg/ml) or an equivalent volume of ethanol (as control) for 6 hr. Cells were then washed once, treated with Alexa Fluor™ 594‐conjugated cholera toxin B subunit (a marker of lipid rafts specific for ganglioside GM1; Sigma‐Aldrich) for 20 min, and subsequently fixed with 4% paraformaldehyde in PBS for 15 min at room temperature. After permeabilization with methanol at −20°C for 5 min, the cells were incubated with TL2.1 for 1 hr, followed by incubation with an FITC‐conjugated anti‐mouse IgG Ab for another 1 hr. The cells were then washed three times with PBS, and images of ganglioside GM1 and TLR2 were obtained using an inverted laser‐scanning microscope A1 (Nikon, Tokyo, Japan).

### 2.7 Statistical analysis

Data are expressed as mean ± standard deviation (*n* = 3). Representative results from more than three separate experiments are shown. Statistical differences were assessed using Student's *t* test. Multiple groups were compared using a two‐tailed one‐way analysis of variance with Dunnett's post hoc test. *p* values < .05 were considered statistically significant.

## 3 RESULTS

### 3.1 Effect of naringenin on inflammatory responses induced through recognition of a synthetic bacterial triacylated lipopeptide, Pam_3_CSK_4_, by TLR2/TLR1

**FIGURE 1:**
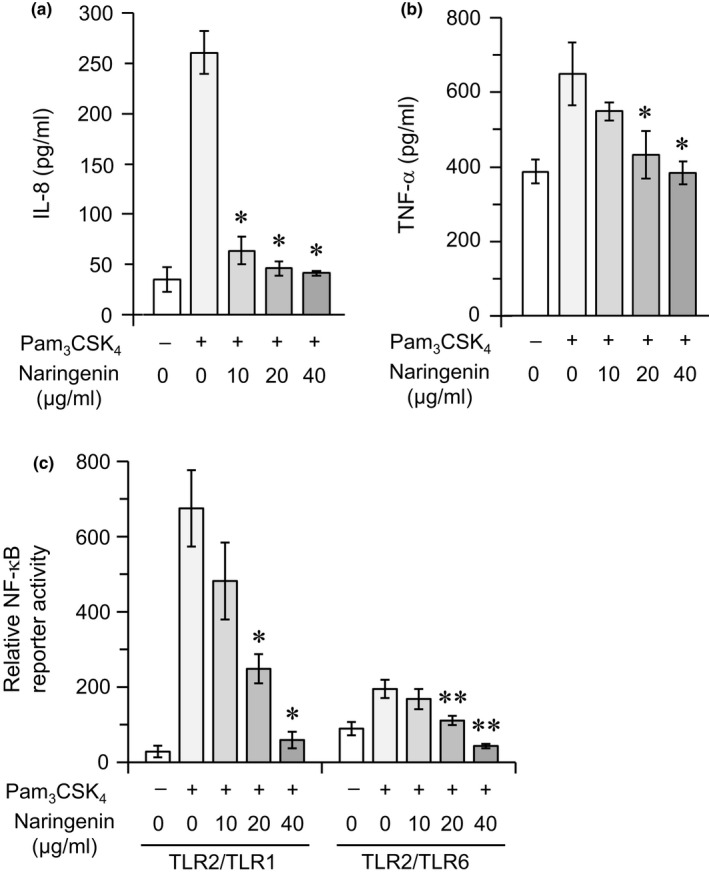
Effect of naringenin on proinflammatory cytokine production and NF‐κB activation in cells stimulated with a synthetic triacylated lipopeptide, Pam_3_CSK_4_. (a, b) THP‐1 cells were stimulated with Pam_3_CSK_4_ (10 μg/ml) in the presence or absence of naringenin (0, 10, 20, or 40 μg/ml) for 6 hr. The levels of IL‐8 (a) and TNF‐α (b) in the culture supernatants were measured using ELISA. Values expressed as mean ± *SD* of triplicate wells are representative of three separate experiments. *p < .05 versus. the culture stimulated with Pam_3_CSK_4_ in the absence of naringenin. (c) HEK293 cells were plated in 24‐well plates and transiently cotransfected with TLR2/TLR1 or TLR2/TLR6 together with an NF‐κB reporter plasmid and Renilla luciferase control reporter plasmid. Cells were stimulated with Pam_3_CSK_4_ (10 μg/ml) in the presence or absence of naringenin (0, 10, 20, or 40 μg/ml) for 6 hr, followed by measurement of relative NF‐κB reporter activity using a luciferase reporter assay. Values expressed as mean ± *SD* of triplicate wells are representative of three separate experiments. *p < .05 versus. the TLR2/TLR1 transfectants stimulated with Pam_3_CSK_4_ in the absence of naringenin; **p < .05 versus. the TLR2/TLR6 transfectants stimulated with Pam_3_CSK_4_ in the absence of naringenin

We first examined the effect of naringenin on the inflammatory responses of human monocytic THP‐1 cells stimulated by a synthetic bacterial triacylated lipopeptide, Pam_3_CSK_4_, which is known to act as a specific ligand for the TLR2/TLR1 heterodimer (Gautam et al., 2006; Jin et al., 2007; Ozinsky et al., 2000). As shown in Figure 1a,b, Pam_3_CSK_4_ stimulated the cells to produce IL‐8 and TNF‐α. Naringenin significantly suppressed these Pam_3_CSK_4_‐induced responses in a dose‐dependent manner (Figure 1a,b). We further examined whether naringenin actually interferes with TLR2/TLR1‐mediated responses using a HEK293 cell line that serves as a nonresponder to most ligands for TLRs, including TLR2, unless appropriately transfected with TLR‐encoding genes (Quevedo‐Diaz et al., 2010).Cellular responses were monitored using an NF‐κB‐driven luciferase reporter. Consistent with previous results (Fujita et al., 2003), HEK293 nontransfectants did not show any response to Pam_3_CSK_4_ (data not shown), whereas TLR2/TLR1 transfectants responded strongly to the stimulus (Figure 1c). TLR2/TLR6 transfectants displayed a weak response to Pam_3_CSK_4_ (Figure 1c), but this response may be mediated through recognition by a TLR2 homodimer and not by a TLR2/TLR6 heterodimer (Fujita et al., 2003). In both cases, naringenin significantly suppressed Pam_3_CSK_4_‐induced responses in a dose‐dependent manner (Figure 1c). These results indicate that naringenin can downregulate inflammatory responses induced through the recognition of Pam_3_CSK_4_ by TLR2/TLR1.

### 3.2 Effect of naringenin on inflammatory responses induced through recognition of a synthetic mycoplasmal diacylated lipopeptide, FSL‐1, by TLR2/TLR6

**FIGURE 2:**
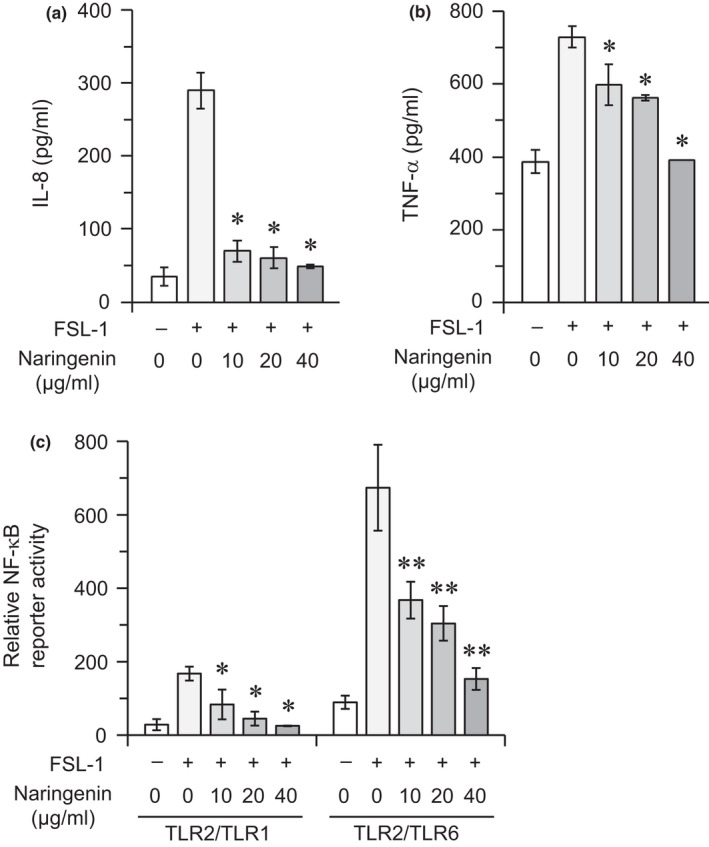
Effect of naringenin on proinflammatory cytokine production and NF‐κB activation in cells stimulated with a synthetic diacylated lipopeptide, FSL‐1. (a, b) THP‐1 cells were stimulated with FSL‐1 (10 nM) in the presence or absence of naringenin (0, 10, 20, or 40 μg/ml) for 6 hr. The levels of IL‐8 (a) and TNF‐α (b) in the culture supernatants were measured using ELISA. Values expressed as mean ± *SD* of triplicate wells are representative of three separate experiments. *p < .05 versus. the culture stimulated with FSL‐1 in the absence of naringenin. (c) HEK293 cells were plated in 24‐well plates and transiently cotransfected with TLR2/TLR1 or TLR2/TLR6 together with the NF‐κB reporter plasmid and Renilla luciferase control reporter plasmid. Cells were stimulated with FSL‐1 (10 nM) in the presence or absence of naringenin (0, 10, 20, or 40 μg/ml) for 6 hr, followed by measurement of the relative NF‐κB reporter activity using a luciferase reporter assay. Values expressed as mean ± *SD* of triplicate wells are representative of three separate experiments. *p < .05 versus. the TLR2/TLR1 transfectants stimulated with FSL‐1 in the absence of naringenin; **p < .05 versus. the TLR2/TLR6 transfectants stimulated with FSL‐1 in the absence of naringenin

We next examined the effect of naringenin on the responses of THP‐1 cells stimulated by a synthetic mycoplasmal diacylated lipopeptide, FSL‐1, which is known to act as a specific ligand for the TLR2/TLR6 heterodimer (Fujita et al., 2003). Similar to Pam_3_CSK_4_, FSL‐1 stimulated the cells to produce IL‐8 and TNF‐α. Naringenin significantly suppressed these FSL‐1‐induced responses in a dose‐dependent manner (Figure 2a,b). Furthermore, FSL‐1 did not stimulate any response in the HEK293 nontransfectants (data not shown), whereas TLR2/TLR6 transfectants strongly responded to the stimulus (Figure 2c). TLR2/TLR1 transfectants displayed a weak response to FSL‐1 (Figure 2c), but this response may be mediated through recognition by a TLR2 homodimer similar to Pam_3_CSK_4_ (Nakata et al., 2006). Naringenin significantly suppressed FSL‐1‐induced responses in a dose‐dependent manner (Figure 2c). These results indicate that naringenin can downregulate inflammatory responses induced through the recognition of FSL‐1 by TLR2/TLR6.

### 3.3 Effect of naringenin on inflammatory responses induced through recognition of a *S. aureus*‐derived crude lipophilic compound by TLR2

**FIGURE 3:**
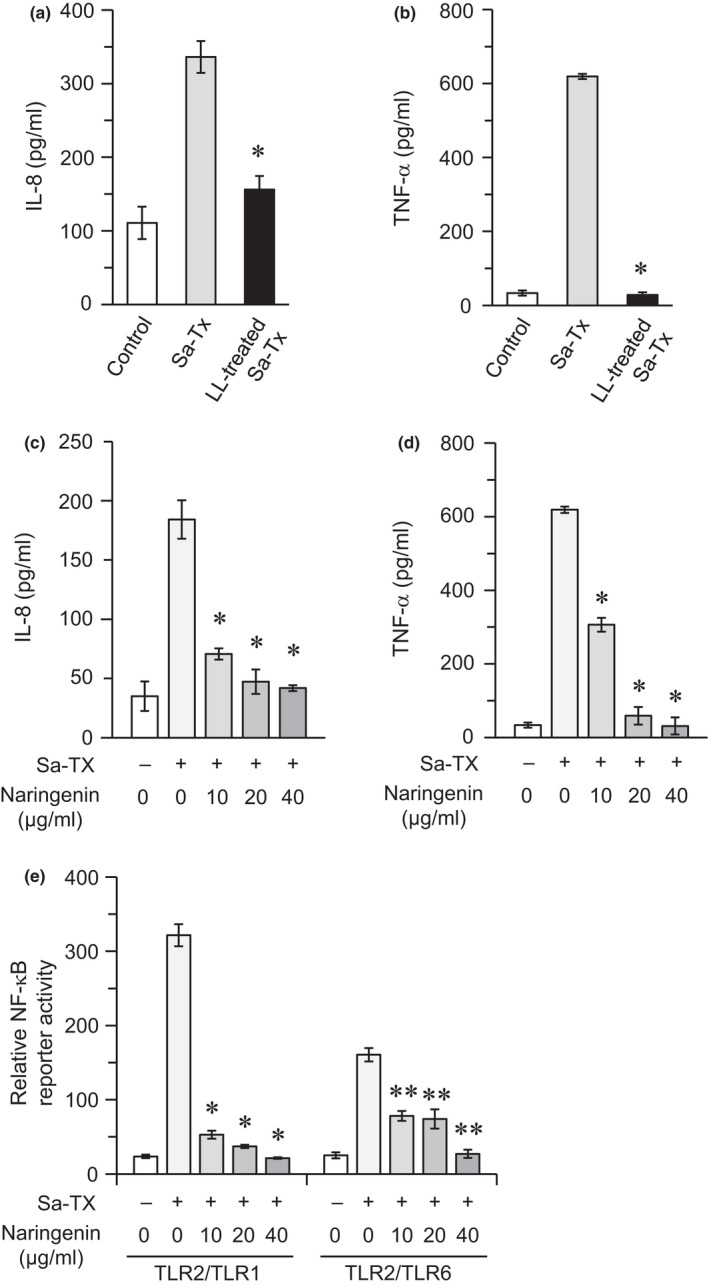
Effect of naringenin on proinflammatory cytokine production and NF‐κB activation in cells stimulated with a TX‐soluble crude lipophilic fraction from *S. aureus*. (a, b) Sa‐TX was treated with lipoprotein lipase (LL). THP‐1 cells were stimulated with Sa‐TX or LL‐treated Sa‐TX for 6 hr. The levels of IL‐8 (a) and TNF‐α (b) in the culture supernatants were measured using ELISA. Values expressed as mean ± *SD* of triplicate wells are representative of three separate experiments. *p < .05 versus. the culture stimulated with nontreated Sa‐TX. (c, d) THP‐1 cells were stimulated with Sa‐TX (1 μg/ml) in the presence or absence of naringenin (0, 10, 20, or 40 μg/ml) for 6 hr. The levels of IL‐8 (a) and TNF‐α (b) in the culture supernatants were measured using ELISA. Values expressed as mean ± *SD* of triplicate wells are representative of three separate experiments. *p < .05 versus. the culture stimulated with Sa‐TX in the absence of naringenin. (e) HEK293 cells were plated in 24‐well plates and transiently cotransfected with TLR2/TLR1 or TLR2/TLR6 together with the NF‐κB reporter plasmid and Renilla luciferase control reporter plasmid. Cells were stimulated with Sa‐TX (1 μg/ml) in the presence or absence of naringenin (0, 10, 20, or 40 μg/ml) for 6 hr, followed by measurement of relative NF‐κB reporter activity using luciferase reporter assay. Values expressed as mean ± *SD* of triplicate wells are representative of three separate experiments. *p < .05 versus. the TLR2/TLR1 transfectants stimulated with Sa‐TX in the absence of naringenin; **p < .05 versus. the TLR2/TLR6 transfectants stimulated with Sa‐TX in the absence of naringenin

In addition to synthetic lipopeptides, previous reports have demonstrated that TLR2 can recognize various types of bacterial compounds including lipoproteins, lipoteichoic acids, and peptidoglycans (Oliveira‐Nascimento et al., 2012). Therefore, we prepared a crude lipophilic extract from *S. aureus*, the cell walls of which contain TLR2‐stimulatory components in abundance (Kang et al., 2015), and designated this preparation as Sa‐TX. As shown in Figure 3a,b, Sa‐TX stimulated THP‐1 cells to produce IL‐8 and TNF‐α. Treatment of Sa‐TX with lipoprotein lipase almost completely attenuated these stimulatory activities (Figure 3a,b), indicating that lipoproteins, and not any other constituents, are responsible for the stimulatory activity of Sa‐TX. *S. aureus*‐derived lipoproteins are reported to be recognized by both TLR2/TLR1 and TLR2/TLR6 (Nguyen et al., 2017).

We examined the effect of naringenin on the responses of cells stimulated with Sa‐TX. In THP‐1 cells, naringenin significantly suppressed Sa‐TX‐induced cytokine production in a dose‐dependent manner (Figure 3c,d). In HEK293 transfectants, Sa‐TX did not stimulate the nontransfectants (data not shown), but strongly stimulated TLR2/TLR1 transfectants (Figure 3c). Sa‐TX also stimulated TLR2/TLR6 transfectants, in a manner similar to Pam_3_CSK_4_, but the response was considerably weak (Figure 3c). Naringenin significantly suppressed these Sa‐TX‐induced responses in a dose‐dependent manner (Figure 3c). These results indicate that naringenin can downregulate inflammatory responses induced through the recognition of crude bacterial compounds by TLR2 complexes.

### 3.4 Naringenin suppresses TLR2‐mediated inflammatory responses through inhibition of receptor clustering on lipid rafts

**FIGURE 4:**
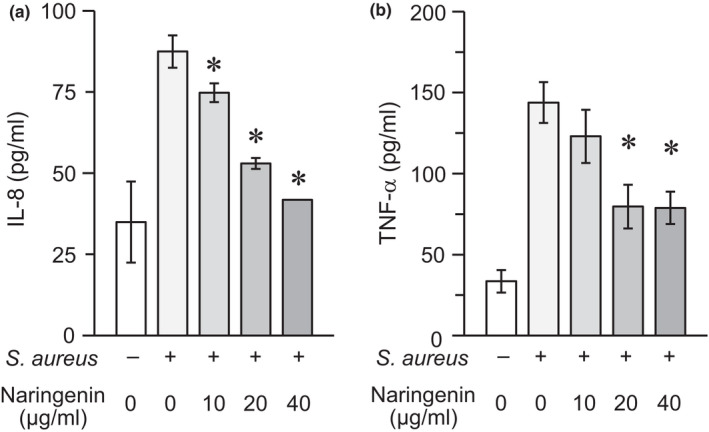
Effect of naringenin on the inflammatory cytokine production in THP‐1 cells stimulated with *S. aureus* cells. THP‐1 cells were stimulated with *S. aureus* cells in the presence or absence of naringenin (0, 10, 20, or 40 μg/ml) for 6 hr. The levels of IL‐8 (a) and TNF‐α (b) in the culture supernatants were measured using ELISA. Values expressed as mean ± *SD* of triplicate wells are representative of three separate experiments. *p < .05 versus. the culture stimulated with *S. aureus* cells in the absence of naringenin

The results described above suggest that naringenin suppresses TLR2‐mediated responses, but its effect does not seem to be specifically exerted on the recognition of distinctive ligands by TLR2. In fact, we found that THP‐1 cells responded to live *S. aureus* cells, which are known to stimulate TLR2‐mediated responses more preferentially than those induced by other innate immune receptors (Musilova et al., 2019), to produce IL‐8 and TNF‐α, and that naringenin could significantly suppress these responses (Figure 4a,b). Thus, naringenin may inhibit the process of TLR2 clustering on the cell membrane and its subsequent signal transduction, but not the interaction between TLR2 and its cognate ligands.

**FIGURE 5:**
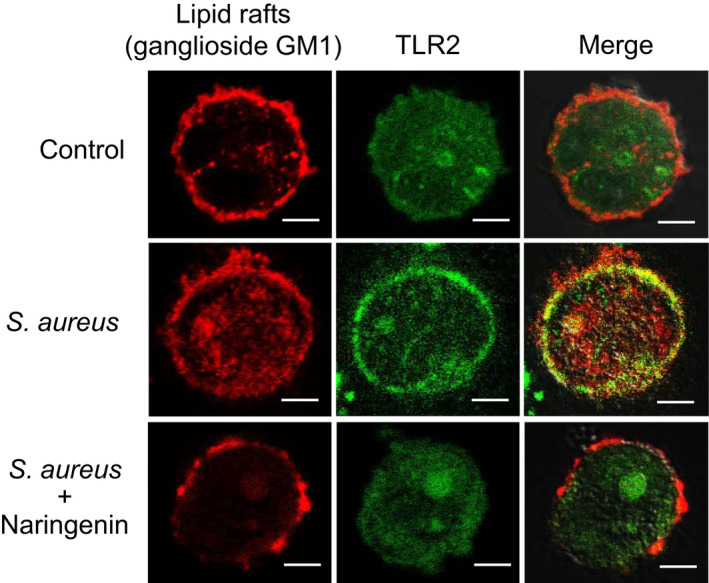
Effect of naringenin on TLR2 recruitment into lipid rafts after stimulation with *S. aureus* cells. THP‐1 cells were stimulated with *S. aureus* cells in the presence or absence of naringenin (40 μg/ml) for 6 hr. Cells were double‐stained for ganglioside GM1 (red) and TLR2 (green). Fluorescence images were obtained using a confocal laser‐scanning microscope. Overlay images are shown in the right panels. Data are representative of three independent experiments with similar results. (Scale bars, 10 μm.)

During the ligand recognition process, cell surface TLR2 is mobilized onto apical lipid rafts, resulting in its accumulation to form lipid raft receptor complexes that can activate signaling (Hellwing et al., 2018). Therefore, we examined the possibility that naringenin interferes with TLR2 clustering on lipid rafts at the cell surface. We visualized ganglioside GM1, a common marker of lipid rafts, and TLR2 in THP‐1 cells stimulated with *S. aureus* using laser‐scanning confocal microscopy. As shown in the nonstimulated control cells in Figure 5, TLR2 localized sparsely on the cell membrane and did not colocalize with lipid rafts. However, in cells stimulated with *S. aureus*, TLR2 was clearly mobilized into and colocalized with lipid rafts (Figure 5). On the other hand, naringenin treatment interfered with TLR2 mobilization and its colocalization with lipid rafts (Figure 5). These results indicate that naringenin inhibits TLR2‐mediated inflammatory responses by interfering with the mobilization and clustering of TLR2 on lipid rafts.

## 4 DISCUSSION

In this study, we demonstrated that naringenin possesses a novel anti‐inflammatory property that can be elicited against TLR2 ligand‐stimulated cells. It mediates its effects by inhibiting TLR2 clustering in lipid rafts on the cell membrane. Our results indicate that the anti‐inflammatory activity of naringenin is effective in ligand recognition by both TLR2/TLR1 and TLR2/TLR6. As TLR2 causes excessive sterile inflammation or exacerbates inflammation through the recognition of DAMPs, naringenin can be an effective therapeutic agent for suppressing such harmful responses.

Naringenin has previously been reported to have anti‐inflammatory effects such as reduction of proinflammatory cytokine production during inflammatory states (Yilma et al., 2013). Several recent studies have investigated the intracellular mechanism of action of naringenin involved in eliciting anti‐inflammatory activities. Jin *et al*. reported that naringenin does not affect the synthesis of the proinflammatory cytokine TNF‐α per se, but inhibits the Golgi‐to‐cell surface delivery of the cytokine (Jin et al., 2017). Additionally, Liu *et al*. found that naringenin inhibits TLR4‐mediated NF‐κB activation through adenosine monophosphate‐activated protein kinase (AMPK)‐dependent upregulation of activating transcription factor 3 (Liu et al., 2016). A more recent report indicates that naringenin activates AMPK‐dependent promotion of autophagy (Ahsan et al., 2020), which is known to suppress inflammatory responses (Zhang et al., 2020). These observations suggest that naringenin affects intracellular protein transport systems or signal transduction. However, it remains unclear whether naringenin modulates cell surface receptor function to elicit anti‐inflammatory activity despite recent reports that show a naringenin‐mediated reduction in TLR2 expression in macrophages and adipocytes (Yilma et al., 2013; Yoshida et al., 2013) and in gestational diabetes mellitus (GDM) model mice (Li et al., 2019). Thus, this is the first report demonstrating the effect of naringenin on the function of TLR2 in cell surface ligand recognition, subsequently resulting in the induction of inflammatory responses.

Lipid rafts are membrane microdomains composed of cholesterol, sphingomyelin, and various membrane proteins (Rietveld & Simons, 1998). Accumulating evidence indicates an important role of lipid rafts in the initiation of immune responses that serve as signal transduction platforms for various cytokine receptors and innate immune PRRs (Gautam et al., 2006; Into et al., 2007; Soong et al., 2004; Triantafilou et al., 2006). In fact, lipid rafts serve as platforms of signal transduction for TLR2 and TLR4 (Triantafilou et al., 2007; Triantafilou et al., 2004; Wong et al., 2009). Additionally, although several flavonoids have a suppressive effect on the functioning of lipid rafts (Kao et al., 2010), it is largely unclear how this effect of flavonoids affects innate immune responses. Therefore, in the present study, we focused on lipid rafts to investigate the effects of naringenin on the functions of TLR2. We demonstrate that cell surface TLR2 does not accumulate with ganglioside GM1, a marker of lipid rafts, in the presence of naringenin, despite the presence of a TLR2 stimulus. This indicates that naringenin inhibits TLR2 clustering to form a signal‐transducing complex on the cell surface. Naringenin is reported to interact with the cell membrane and to decrease membrane fluidity in the hydrophobic region of the lipid bilayer (Arora et al., 2000). Such decreased membrane fluidity may inhibit the accumulation of TLR2 and its associated molecules in lipid rafts. Moreover because previous reports indicate that the functioning of lipid rafts can be suppressed by antioxidants such as vitamin E (DiPasquale et al., 2020), the antioxidative property of naringenin may be involved in this effect and should be investigated further in future studies.

In summary, the findings of the present study suggest a novel property of naringenin in the suppression of inflammatory responses in TLR2 ligand‐stimulated cells through inhibition of TLR2 clustering in lipid rafts. Such an effect can be elicited against TLR2 heterodimers formed with TLR1 and TLR6. Thus, naringenin may serve as a useful new therapeutic agent against excessive inflammation caused by severe microbial infections, inflammatory diseases, and autoimmune diseases.

## 6 CONFLICT OF INTEREST

The authors declare no conflicts of interest associated with this manuscript.

## 7 ETHICAL APPROVAL

This study does not involve any human or animal testing.

## References

[fsn32063-bib-0001] Ahsan, A. U. , Sharma, V. L. , Wani, A. , & Chopra, M. (2020). Naringenin upregulates AMPK‐mediated autophagy to rescue neuronal cells From β‐amyloid ((1–42)) evoked neurotoxicity. Molecular Neurobiology, 57(8), 3589–3602. 10.1007/s12035-020-01969-4 32542594

[fsn32063-bib-0002] Akira, S. , & Takeda, K. (2004). Toll‐like receptor signalling. Nature Reviews Immunology, 4(7), 499–511. 10.1038/nri1391 15229469

[fsn32063-bib-0003] Aliprantis, A. O. , Yang, R. B. , Mark, M. R. , Suggett, S. , Devaux, B. , Radolf, J. D. , & Zychlinsky, A. (1999). Cell activation and apoptosis by bacterial lipoproteins through toll‐like receptor‐2. Science, 285(5428), 736–739. 10.1126/science.285.5428.736 10426996

[fsn32063-bib-0004] Arora, A. , Byrem, T. M. , Nair, M. G. , & Strasburg, G. M. (2000). Modulation of liposomal membrane fluidity by flavonoids and isoflavonoids. Archives of Biochemistry and Biophysics, 373(1), 102–109. 10.1006/abbi.1999.1525 10620328

[fsn32063-bib-0005] DiPasquale, M. , Nguyen, M. H. L. , Rickeard, B. W. , Cesca, N. , Tannous, C. , Castillo, S. R. , Katsaras, J. , Kelley, E. G. , Heberle, F. A. , & Marquardt, D. (2020). The antioxidant vitamin E as a membrane raft modulator: Tocopherols do not abolish lipid domains. Biochimica et Biophysica Acta Biomembranes, 1862(8), 183189 10.1016/j.bbamem.2020.183189 31954106 PMC10443432

[fsn32063-bib-0006] Felgines, C. , Texier, O. , Morand, C. , Manach, C. , Scalbert, A. , Régerat, F. , & Rémésy, C. (2000). Bioavailability of the flavanone naringenin and its glycosides in rats. American Journal of Physiology‐Gastrointestinal and Liver Physiology, 279(6), G1148–1154. 10.1152/ajpgi.2000.279.6.G1148 11093936

[fsn32063-bib-0007] Fischer, M. , & Ehlers, M. (2008). Toll‐like receptors in autoimmunity. Annals of the New York Academy of Sciences, 1143, 21–34. 10.1196/annals.1443.012 19076342

[fsn32063-bib-0008] Fujita, M. , Into, T. , Yasuda, M. , Okusawa, T. , Hamahira, S. , Kuroki, Y. , Eto, A. , Nisizawa, T. , Morita, M. , & Shibata, K. (2003). Involvement of leucine residues at positions 107, 112, and 115 in a leucine‐rich repeat motif of human Toll‐like receptor 2 in the recognition of diacylated lipoproteins and lipopeptides and *Staphylococcus aureus* peptidoglycans. Journal of Immunology, 171(7), 3675–3683. 10.4049/jimmunol.171.7.3675 14500665

[fsn32063-bib-0009] Gautam, J. K. , Ashish, XXX. , Comeau, L. D. , Krueger, J. K. , & Smith, M. F. (2006). Structural and functional evidence for the role of the TLR2 DD loop in TLR1/TLR2 heterodimerization and signaling. Journal of Biological Chemistry, 281(40), 30132–30142. 10.1074/jbc.M602057200 PMC1769446 16893894

[fsn32063-bib-0010] Ghofrani, S. , Joghataei, M. T. , Mohseni, S. , Baluchnejadmojarad, T. , Bagheri, M. , Khamse, S. , & Roghani, M. (2015). Naringenin improves learning and memory in an Alzheimer's disease rat model: Insights into the underlying mechanisms. European Journal of Pharmacology, 764, 195–201. 10.1016/j.ejphar.2015.07.001 26148826

[fsn32063-bib-0011] Gutiérrez‐Venegas, G. , Kawasaki‐Cárdenas, P. , Arroyo‐Cruz, S. R. , & Maldonado‐Frías, S. (2006). Luteolin inhibits lipopolysaccharide actions on human gingival fibroblasts. European Journal of Pharmacology, 541(1–2), 95–105. 10.1016/j.ejphar.2006.03.069 16762341

[fsn32063-bib-0012] Hashimoto, M. , Tawaratsumida, K. , Kariya, H. , Aoyama, K. , Tamura, T. , & Suda, Y. (2006). Lipoprotein is a predominant Toll‐like receptor 2 ligand in *Staphylococcus aureus* cell wall components. International Immunology, 18(2), 355–362. 10.1093/intimm/dxh374 16373361

[fsn32063-bib-0013] Hellwing, C. , Schoeniger, A. , Roessler, C. , Leimert, A. , & Schumann, J. (2018). Lipid raft localization of TLR2 and its co‐receptors is independent of membrane lipid composition. PeerJ, 6, e4212 10.7717/peerj.4212 29312832 PMC5757419

[fsn32063-bib-0014] Into, T. , Dohkan, J. , Inomata, M. , Nakashima, M. , Shibata, K. , & Matsushita, K. (2007). Synthesis and characterization of a dipalmitoylated lipopeptide derived from paralogous lipoproteins of Mycoplasma pneumoniae. Infection and Immunity, 75(5), 2253–2259. 10.1128/iai.00141-07 17325056 PMC1865785

[fsn32063-bib-0015] Jin, L. , Zeng, W. , Zhang, F. , Zhang, C. , & Liang, W. (2017). Naringenin ameliorates acute inflammation by regulating intracellular cytokine degradation. Journal of Immunology, 199(10), 3466–3477. 10.4049/jimmunol.1602016 28993518

[fsn32063-bib-0016] Jin, M. S. , Kim, S. E. , Heo, J. Y. , Lee, M. E. , Kim, H. M. , Paik, S.‐G. , Lee, H. , & Lee, J.‐O. (2007). Crystal structure of the TLR1‐TLR2 heterodimer induced by binding of a tri‐acylated lipopeptide. Cell, 130(6), 1071–1082. 10.1016/j.cell.2007.09.008 17889651

[fsn32063-bib-0017] Kang, S. S. , Noh, S. Y. , Park, O. J. , Yun, C. H. , & Han, S. H. (2015). *Staphylococcus aureus* induces IL‐8 expression through its lipoproteins in the human intestinal epithelial cell, Caco‐2. Cytokine, 75(1), 174–180. 10.1016/j.cyto.2015.04.017 25982554

[fsn32063-bib-0018] Kao, T. K. , Ou, Y. C. , Raung, S. L. , Lai, C. Y. , Liao, S. L. , & Chen, C. J. (2010). Inhibition of nitric oxide production by quercetin in endotoxin/cytokine‐stimulated microglia. Life Sciences, 86(9–10), 315–321. 10.1016/j.lfs.2009.12.014 20060843

[fsn32063-bib-0019] Kataoka, H. , Taniguchi, M. , Fukamachi, H. , Arimoto, T. , Morisaki, H. , & Kuwata, H. (2014). *Rothia dentocariosa* induces TNF‐alpha production in a TLR2‐dependent manner. Pathogens and Disease, 71(1), 65–68. 10.1111/2049-632x.12115 24265267

[fsn32063-bib-0020] Kawaguchi, K. , Kikuchi, S. , Hasunuma, R. , Maruyama, H. , Ryll, R. , & Kumazawa, Y. (2004). Suppression of infection‐induced endotoxin shock in mice by a citrus flavanone naringin. Planta Medica, 70(1), 17–22. 10.1055/s-2004-815449 14765287

[fsn32063-bib-0021] Li, S. , Zhang, Y. , Sun, Y. , Zhang, G. , Bai, J. , Guo, J. , Su, X. , Du, H. , Cao, X. , Yang, J. , & Wang, T. (2019). Naringenin improves insulin sensitivity in gestational diabetes mellitus mice through AMPK. Nutrition & Diabetes, 9(28). 10.1038/s41387-019-0095-8 PMC6779739 31591391

[fsn32063-bib-0022] Liu, X. , Wang, N. , Fan, S. , Zheng, X. , Yang, Y. , Zhu, Y. , Lu, Y. , Chen, Q. , Zhou, H. , & Zheng, J. (2016). The citrus flavonoid naringenin confers protection in a murine endotoxaemia model through AMPK‐ATF3‐dependent negative regulation of the TLR4 signalling pathway. Scientific Reports, 6, 39735 10.1038/srep39735 28004841 PMC5177915

[fsn32063-bib-0023] Musilova, J. , Mulcahy, M. E. , Kuijk, M. M. , McLoughlin, R. M. , & Bowie, A. G. (2019). Toll‐like receptor 2‐dependent endosomal signaling by *Staphylococcus aureus* in monocytes induces type I interferon and promotes intracellular survival. Journal of Biological Chemistry, 294(45), 17031–17042. 10.1074/jbc.RA119.009302 PMC6851302 31558608

[fsn32063-bib-0024] Nakata, T. , Yasuda, M. , Fujita, M. , Kataoka, H. , Kiura, K. , Sano, H. , & Shibata, K. (2006). CD14 directly binds to triacylated lipopeptides and facilitates recognition of the lipopeptides by the receptor complex of Toll‐like receptors 2 and 1 without binding to the complex. Cellular Microbiology, 8(12), 1899–1909. 10.1111/j.1462-5822.2006.00756.x 16848791

[fsn32063-bib-0025] Nguyen, M.‐T. , Uebele, J. , Kumari, N. , Nakayama, H. , Peter, L. , Ticha, O. , Woischnig, A.‐K. , Schmaler, M. , Khanna, N. , Dohmae, N. , Lee, B. L. , Bekeredjian‐Ding, I. , & Götz, F. (2017). Lipid moieties on lipoproteins of commensal and non‐commensal staphylococci induce differential immune responses. Nature Communications, 8(1), 2246 10.1038/s41467-017-02234-4 PMC5740139 29269769

[fsn32063-bib-0026] Oliveira‐Nascimento, L. , Massari, P. , & Wetzler, L. M. (2012). The role of TLR2 in infection and immunity. Frontiers in Immunology, 3, 79 10.3389/fimmu.2012.00079 22566960 PMC3342043

[fsn32063-bib-0027] Olszanecki, R. , Gebska, A. , Kozlovski, V. I. , & Gryglewski, R. J. (2002). Flavonoids and nitric oxide synthase. Journal of Physiology and Pharmacology, 53(4 Pt 1), 571–584. 12512693

[fsn32063-bib-0028] Ozinsky, A. , Underhill, D. M. , Fontenot, J. D. , Hajjar, A. M. , Smith, K. D. , Wilson, C. B. , Schroeder, L. , & Aderem, A. (2000). The repertoire for pattern recognition of pathogens by the innate immune system is defined by cooperation between toll‐like receptors. Proceedings of National Academy of Sciences of the United States of America, 97(25), 13766–13771. 10.1073/pnas.250476497 PMC17650 11095740

[fsn32063-bib-0029] Quevedo‐Diaz, M. A. , Song, C. , Xiong, Y. , Chen, H. , Wahl, L. M. , Radulovic, S. , & Medvedev, A. E. (2010). Involvement of TLR2 and TLR4 in cell responses to *Rickettsia akari* . Journal of Leukocyte Biology, 88(4), 675–685. 10.1189/jlb.1009674 20616112 PMC2974430

[fsn32063-bib-0030] Rietveld, A. , & Simons, K. (1998). The differential miscibility of lipids as the basis for the formation of functional membrane rafts. Biochimica Et Biophysica Acta (BBA) ‐ Bioenergetics, 1376(3), 467–479. 10.1016/s0304-4157(98)00019-7 9805010

[fsn32063-bib-0031] Santangelo, C. , Varì, R. , Scazzocchio, B. , Di Benedetto, R. , Filesi, C. , & Masella, R. (2007). Polyphenols, intracellular signalling and inflammation. Annali dell'istituto Superiore di Sanita, 43(4), 394–405. 18209273

[fsn32063-bib-0032] Shibata, K. , Hasebe, A. , Into, T. , Yamada, M. , & Watanabe, T. (2000). The N‐terminal lipopeptide of a 44‐kDa membrane‐bound lipoprotein *of Mycoplasma salivarium* is responsible for the expression of intercellular adhesion molecule‐1 on the cell surface of normal human gingival fibroblasts. Journal of Immunology, 165(11), 6538–6544. 10.4049/jimmunol.165.11.6538 11086096

[fsn32063-bib-0033] Shimazu, R. , Akashi, S. , Ogata, H. , Nagai, Y. , Fukudome, K. , Miyake, K. , & Kimoto, M. (1999). MD‐2, a molecule that confers lipopolysaccharide responsiveness on Toll‐like receptor 4. Journal of Experimental Medicine, 189(11), 1777–1782. 10.1084/jem.189.11.1777 PMC2193086 10359581

[fsn32063-bib-0034] Soong, G. , Reddy, B. , Sokol, S. , Adamo, R. , & Prince, A. (2004). TLR2 is mobilized into an apical lipid raft receptor complex to signal infection in airway epithelial cells. Journal of Clinical Investigation, 113(10), 1482–1489. 10.1172/jci20773 PMC406530 15146246

[fsn32063-bib-0035] Takeda, K. , Kaisho, T. , & Akira, S. (2003). Toll‐like receptors. Annual Review of Immunology, 21, 335–376. 10.1146/annurev.immunol.21.120601.141126 12524386

[fsn32063-bib-0036] Takeuchi, O. , Kawai, T. , Mühlradt, P. F. , Morr, M. , Radolf, J. D. , Zychlinsky, A. , Takeda, K. , & Akira, S. (2001). Discrimination of bacterial lipoproteins by Toll‐like receptor 6. International Immunology, 13(7), 933–940. 10.1093/intimm/13.7.933 11431423

[fsn32063-bib-0037] Takeuchi, O. , Sato, S. , Horiuchi, T. , Hoshino, K. , Takeda, K. , Dong, Z. , Modlin, R. L. , & Akira, S. (2002). Cutting edge: Role of Toll‐like receptor 1 in mediating immune response to microbial lipoproteins. Journal of Immunology, 169(1), 10–14. 10.4049/jimmunol.169.1.10 12077222

[fsn32063-bib-0038] Triantafilou, M. , Gamper, F. G. , Haston, R. M. , Mouratis, M. A. , Morath, S. , Hartung, T. , & Triantafilou, K. (2006). Membrane sorting of toll‐like receptor (TLR)‐2/6 and TLR2/1 heterodimers at the cell surface determines heterotypic associations with CD36 and intracellular targeting. Journal of Biological Chemistry, 281(41), 31002–31011. 10.1074/jbc.M602794200 16880211

[fsn32063-bib-0039] Triantafilou, M. , Gamper, F. G. J. , Lepper, P. M. , Mouratis, M. A. , Schumann, C. , Harokopakis, E. , Schifferle, R. E. , Hajishengallis, G. , & Triantafilou, K. (2007). Lipopolysaccharides from atherosclerosis‐associated bacteria antagonize TLR4, induce formation of TLR2/1/CD36 complexes in lipid rafts and trigger TLR2‐induced inflammatory responses in human vascular endothelial cells. Cellular Microbiology, 9(8), 2030–2039. 10.1111/j.1462-5822.2007.00935.x 17419716

[fsn32063-bib-0040] Triantafilou, M. , Morath, S. , Mackie, A. , Hartung, T. , & Triantafilou, K. (2004). Lateral diffusion of Toll‐like receptors reveals that they are transiently confined within lipid rafts on the plasma membrane. Journal of Cell Science, 117(Pt 17), 4007–4014. 10.1242/jcs.01270 15286178

[fsn32063-bib-0041] Uzel, A. , Sorkun, K. , Onçağ, O. , Cogŭlu, D. , Gençay, O. , & Salih, B. (2005). Chemical compositions and antimicrobial activities of four different *Anatolian propolis* samples. Microbiological Research, 160(2), 189–195. 10.1016/j.micres.2005.01.002 15881836

[fsn32063-bib-0042] van Zoelen, M. A. D. , Yang, H. , Florquin, S. , Meijers, J. C. M. , Akira, S. , Arnold, B. , Nawroth, P. P. , Bierhaus, A. , Tracey, K. J. , & Poll, T. V. D. (2009). Role of toll‐like receptors 2 and 4, and the receptor for advanced glycation end products in high‐mobility group box 1‐induced inflammation *in vivo* . Shock, 31(3), 280–284. 10.1097/SHK.0b013e318186262d 19218854 PMC4535325

[fsn32063-bib-0043] Wong, S. W. , Kwon, M. J. , Choi, A. M. , Kim, H. P. , Nakahira, K. , & Hwang, D. H. (2009). Fatty acids modulate Toll‐like receptor 4 activation through regulation of receptor dimerization and recruitment into lipid rafts in a reactive oxygen species‐dependent manner. Journal of Biological Chemistry, 284(40), 27384–27392. 10.1074/jbc.M109.044065 PMC2785667 19648648

[fsn32063-bib-0044] Yilma, A. N. , Singh, S. R. , Morici, L. , & Dennis, V. A. (2013). Flavonoid naringenin: A potential immunomodulator for *Chlamydia trachomatis* inflammation. Mediators of Inflammation, 2013, 102457 10.1155/2013/102457 23766556 PMC3676976

[fsn32063-bib-0045] Yoshida, H. , Watanabe, W. , Oomagari, H. , Tsuruta, E. , Shida, M. , & Kurokawa, M. (2013). Citrus flavonoid naringenin inhibits TLR2 expression in adipocytes. The Journal of Nutritional Biochemistry, 24(7), 1276–1284. 10.1016/j.jnutbio.2012.10.003 23333096

[fsn32063-bib-0046] Zaragozá, C. , Villaescusa, L. , Monserrat, J. , Zaragozá, F. , & Álvarez‐Mon, M. (2020). Potential therapeutic anti‐inflammatory and immunomodulatory effects of dihydroflavones, flavones, and flavonols. Molecules, 25(4), 1017 10.3390/molecules25041017 PMC7070238 32102475

[fsn32063-bib-0047] Zhang, X. , Liang, T. , Yang, W. , Zhang, L. , Wu, S. , Yan, C. , & Li, Q. (2020). *Astragalus membranaceus* injection suppresses production of interleukin‐6 by activating autophagy through the AMPK‐mTOR pathway in lipopolysaccharide‐stimulated macrophages. Oxidative Medicine and Cellular Longevity, 2020, 1364147 10.1155/2020/1364147 32724488 PMC7364262

